# The impacts of different dietary restriction regimens on aging and longevity: from yeast to humans

**DOI:** 10.1186/s12929-025-01188-w

**Published:** 2025-10-03

**Authors:** Tsui-Ting Ching, Ao-Lin Hsu

**Affiliations:** 1 Institute of Biopharmaceutical Sciences, National Yang Ming Chiao Tung University, Taipei, Taiwan; 2 Institute of Biochemistry and Molecular Biology, National Yang Ming Chiao Tung University, Taipei, Taiwan; 3 Institute of Molecular Biology, Academia Sinica, Taipei, Taiwan

**Keywords:** Dietary restriction, Caloric restriction, Protein restriction, Intermittent fasting, Time-restricted fasting, Longevity and aging, Metabolic reprogramming, Nutrient-sensing, Multiple model organisms, Anti-aging interventions

## Abstract

Dietary restriction (DR) refers to a broad set of interventions that limit the intake of specific nutrients or overall food consumption, either in quantity or timing, without causing malnutrition. DR has long been considered the most robust intervention for increasing healthspan and lifespan. This includes, not exhaustively, caloric restriction (CR), protein restriction (PR), amino acid restriction (AAR), intermittent fasting (IF), and time-restricted fasting (TRF), each with overlapping but distinct metabolic and physiological effects. This brief review examines the current scientific understanding of how some of the most commonly employed DR regimens may impact metabolism, lifespan, and healthspan. Particular attention is given to the underlying biological mechanisms and supporting evidence derived from both human clinical studies and fundamental biological research conducted with model organisms ranging from yeast to non-human primates.

## Introduction to dietary restriction

Dietary restriction (DR) refers to a wide range of dietary regimens that limit the intake of certain nutrients or overall food consumption, either in quantity or timing, while ensuring adequate nutrition is maintained. This includes, not exhaustively, caloric restriction (CR), protein restriction (PR), amino acid restriction (AAR), intermittent fasting (IF), and time-restricted fasting (TRF). Among all potential anti-aging interventions, DR has been regarded as the most robust and conserved intervention for increasing healthspan and lifespan across taxa [1]. Initial studies of each dietary restriction regimen in different model organisms are summarized in Table 1.

**Table 1:** List of initial studies of different DR regimens in various model organisms. Here, we list the initial studies of CR, PR, AAR, IF, or TRF in different model organisms that we were able to find. The treatment column indicates the exact dietary regimens used in each study

Type of dietary restriction	Model organism	Treatment	Reference
Caloric restriction (CR)	Yeast	Glucose reduced from 2% to 0.5%	Lin et al., (RLS); Smith et al., (CLS) [8, 9]
	Nematode	Bacterial dilutions from 1x10^10 to 10^8 CFU	Klass et al., [11]
	Fruit fly	35% less yeast and sugar	Chapman et al., [15]
	Rat	25–65% CR	McCay et al., [3]
	Mouse	55–65% CR	Weindruch et al., [18]
	Grey mouse lemur	30% CR	Pifferi et al., [31]
	Rhesus monkey	30% CR	Colman et al., [28]
Protein/amino acid restriction (PR/AAR)	Yeast	Remove all amino acids, except 4 essential a.a.	Jiang et al., [41]
	Yeast	50-90% less methionine	Koc et al., [46]
	Fruit fly	57% less protein (with 28% CR)	Mair et al., [52]
	Fruit fly	67%–89% less methionine	Troen et al., [48]
	Fruit fly	85% less BCAAs	Juricic et al., [49]
	Rat	40% less protein	Yu et al., [55]
	Rat	80% less methionine	Orentreich et al., [56]
	Rat	Tryptophan deficiency	Segall et al., [66]
	Mouse	83% less protein	Stoltzner et al., [54]
	Mouse	67% less BCAAs	Richardson et al., [62]
	Mouse	77% less methionine	Miller et al., [57]
	Mouse	60% less tryptophan	De Marte et al., [64]
Intermittent/time-restricted fasting (IF/TRF)	Nematode	ADF	Honjoh et al., [72]
	Fruit fly	2-day fed:5-day fasted IF	Catterson et al., [73]
	Rat	ADF	Goodrick et al., [78]
	Mouse	ADF	Goodrick et al., [75]
	Mouse	TRF for 8 hr per day	Hatori et al., [84]

### Calorie restriction

Caloric restriction (CR) is a reduction in energy intake below the level of calorie consumption under ad libitum conditions without causing malnutrition. The concept of caloric restriction has been recognized for centuries, with early philosophical and medical texts acknowledging the potential benefits of reduced food intake [2]. Scientific exploration of CR began in the early twentieth century, notably with studies on rats that demonstrated a significant extension of both mean and maximal lifespan through calorie reduction [3]. McCay’s groundbreaking research in the 1930s provided some of the first experimental evidence linking CR to longevity.

Subsequent studies expanded across various model organisms, consistently reaffirming the longevity benefits of CR across multiple species, including yeast, worms, flies, rodents, and primates [4–7]. In yeast, CR typically involves lowering glucose concentrations in the growth medium from 2% to 0.5% or less. This reduction significantly extends both the replicative lifespan (RLS; the number of daughter cells that a yeast mother cell can produce) [8] and the chronological lifespan (CLS; how long non-dividing cells remain viable) [9]. In nematode *Caenorhabditis elegans*, CR extends lifespan by up to 50%, depending on the method used. Approaches include bacterial dilution (reducing food availability) [10, 11] and genetic manipulation (such as *eat-2* mutants with reduced feeding rate) [12]. CR also improves proteostasis and reduces the accumulation of age-related protein aggregates, contributing to improved cellular function and organismal health in worms [13]. In fruit flies, *Drosophila*, studies have observed CR-induced lifespan extension by 30–50%, particularly when CR is initiated early in life [14, 15]. CR has also been shown to preserve physical activity and delay the onset of age-related decline in reproductive functions in flies [16, 17].

In rodents, the impact of CR is typically investigated by reducing calorie intake to around 60–70% of the ad libitum consumption. Reductions of calorie intake at this level extend both mean and maximum lifespan across various rodent species, including mice and rats [3, 18–20]. Rodent studies have also been instrumental in understanding the long-term effects of CR on disease onset. In studies where rodents are subjected to CR, the onset of age-related diseases, such as cancer, cardiovascular disease, and neurodegeneration, is delayed or attenuated [18, 21, 22]. For example, CR has been shown to reduce the incidence and multiplicity of spontaneous and chemically induced tumors in mice and rats, particularly in tissues such as the liver, lung, and mammary glands [23]. Moreover, CR markedly lowers the development of atherosclerosis [24], one major contributor to cardiovascular morbidity in aging populations. In terms of neurodegeneration, CR has been linked to enhanced synaptic plasticity and decreased accumulation of amyloid-beta, a hallmark of Alzheimer’s disease pathology, in mouse models [25–27].

Research on non-human primates, such as rhesus monkeys (*Macaca mulatta*) and grey mouse lemurs (*Microcebus murinus*), has significantly contributed to the understanding of the potential effects of CR on aging and healthspan in humans, as these primates share many physiological and genetic similarities with humans. Results from these studies have demonstrated that CR improved overall health and longevity while delaying the onset of age-related diseases in these primates [28–31]. For example, CR has been linked to a reduced incidence of cancer, cardiovascular diseases, and metabolic disorders [28, 29]. Additionally, it helps preserve brain volume and enhances cognitive function in aged monkeys [28, 32], suggesting its neuroprotective effects.

Research on CR in humans faces challenges due to difficulties in conducting long-term controlled studies. However, evidence gathered from clinical trials, epidemiological research, and observational studies strongly indicates that reducing calorie intake provides significant health advantages [33, 34]. One of the most well-known human studies is the CALERIE (Comprehensive Assessment of Long-term Effects of Reducing Intake of Energy), a 2-year, randomized controlled trial for non-obese individuals [35]. The results showed that moderate caloric restriction (around 25% reduction in daily caloric intake) led to significant improvements in several key biomarkers of aging, such as insulin sensitivity, blood pressure, inflammatory markers, and lipid profiles [36, 37]. Participants in the CALERIE trial also showed improvements in muscle function and immune system performance [38, 39], indicating that CR can have broad, positive effects on tissue maintenance during aging. (All the human clinical studies mentioned here and in the later sections are summarized in Table 2.)

**Table 2:** List of all human clinical studies discussed in the main text

Study	Subject characteristics	Study protocol	Duration	Primary endpoints	Key observations	References
CALERIE trial	Non-obese healthy adults (aged 21-50)	Randomized controlled trial with 25% reduction in daily caloric intake	2 years	Biomarkers of aging, cardiometabolic health	Significant improvements in insulin sensitivity, blood pressure, inflammatory markers, lipid profiles, muscle function, and immune system performance	Meydani et al.,Most et al., Kraus et al., Das et al., [36–39]
NHANES III analysis	Adults aged 50-65 vs 65+	Epidemiological analysis of protein intake levels	Long-term follow-up	Mortality	High protein intake (≥20% of calories) associated with 75% increase in overall and cancer mortality in 50-65 age group; this negative association is not observed in >65 age group	Levine et al., [68]
Plant vs animal protein study	Health care professionals	Observational analysis of protein source	Long-term follow-up	Mortality	Plant-based proteins associated with reduced mortality risk; animal-based protein linked to increased risk	Song et al., [69]
Protein restriction (7%–9% protein diet)	Middle-aged overweight and mildly obese men	Randomized clinical trials of reduced protein intake	43 ± 11days	Glucose metabolism	Reducing protein intake improved glucose homeostasis	Fontana et al., [70]
Low-protein diet study	Lean, healthy men	Low-protein, high-carbohydrate (LPHC; 8 E% protein) diet vs a habitual higher protein (16 E% protein) diet	5 weeks	Insulin sensitivity, metabolic parameters	Improved whole-body insulin sensitivity when proteins replaced by carbohydrates; tendency to consume more calories from fats/carbohydrates to maintain body weight	Nicolaisen et al., [71]
Early time-restricted feeding	Prediabetic men	eTRF (6-hour eating window) vs control schedule (12-hr)	5 weeks	Insulin sensitivity, metabolic markers	Improved insulin sensitivity even without weight loss	Sutton et al., [92]
10-hour time-restricted feeding	Individuals with metabolic syndrome	10-hour time-restricted feeding	2-week baseline and 12-week intervention periods	Metabolic and cardiovascular outcomes	Reduced fasting glucose, lowered HbA1c, improved blood pressure and atherogenic lipid levels	Wilkinson et al., [93]
IF and brain function	Overweight adults aged 55-70	5:2 intermittent fasting (480 Kcal/day for 2 days; USDA diet for 5 days )	8 weeks	Cognitive function, brain aging	Enhanced executive function and memory; attenuated rate of brain aging	Kapogiannis et al., [94]

### Protein/amino acid restriction

Protein restriction (PR) or amino acid restriction (AAR) refers to dietary interventions that lower total protein intake or limit specific amino acids, respectively, while maintaining overall nutritional adequacy. Common PR strategies include lowering daily protein intake, which also leads to reduced caloric intake, or substituting protein content in the diet with carbohydrates without altering the total caloric intake. The longevity effects of PR have been well-documented in several model organisms, including yeast, fruit flies, and rodents [40]. On the other hand, targeting specific amino acids such as methionine, tryptophan, or branched-chain amino acids (BCAAs) like leucine, isoleucine, and valine has also been demonstrated to increase longevity.

In yeast, limiting the availability of all nonessential amino acids [41] or specific amino acids such as methionine, asparagine, or glutamate extends RLS or CLS independently of calorie intake [42–46]. Due to the lack of a precisely defined food source for cultivating *C. elegans* in the laboratory, direct implementation of protein or amino acid restriction has not been feasible. However, indirect evidence from worms cultured on metformin-treated bacteria suggests that methionine restriction may be responsible for the observed longevity phenotype in metformin-treated worms [47]. In *Drosophila*, both protein and amino acid restriction have been shown to extend lifespan, particularly when methionine or BCAA is restricted [48–52]. Grandison et al. demonstrated that reducing dietary protein, while supplementing only methionine, reversed the lifespan-extending effect, highlighting methionine’s central role in aging regulation [53]. In addition to methionine restriction, recent studies have suggested that BCAA restriction may also increase survival in flies [49].

In rodents, reducing dietary protein by 40–83% typically leads to a 10–20% increase in lifespan [54, 55], whereas methionine restriction alone has been shown to extend lifespan by 30–40%, independent of total caloric intake [56–58]. Besides lifespan extension, methionine restriction has been shown to reduce oxidative stress and promote leanness in rodents [59, 60]. Similarly, emerging evidence suggests that restricting BCAAs may promote longevity and improve metabolic profiles in mice [61, 62]. Notably, both protein restriction and BCAA restriction exhibit sex-dependent effects in mice [62, 63]. Finally, dietary restrictions of tryptophan have been reported to extend the lifespan of mice and rats and delay the onset of age-associated diseases in various studies dating back to the 1970s and 1980s [64–66]. Interestingly, a recent study suggests that tryptophan restriction may be an evolutionarily conserved intervention for promoting longevity. The administration of ibuprofen, an inhibitor of tryptophan uptake, extends the lifespan of yeast, worms, and flies [67]. However, the underlying mechanism of how tryptophan restriction may exhibit its longevity benefits remains largely unknown.

Direct evidence linking PR to lifespan extension remains limited in humans. However, accumulating data suggest that reducing total protein intake, particularly from animal sources, and specific amino acids may yield metabolic benefits and promote healthy aging. Epidemiological studies, such as analyses of data from the National Health and Nutrition Examination Survey (NHANES), have shown that adults aged 50–65 consuming high levels of protein (≥ 20% of daily calories) had a 75% increase in overall mortality and a fourfold increase in cancer mortality, compared to those with moderate protein intake [68]. Interestingly, these associations were not observed in individuals over 65, highlighting the importance of age-specific recommendations. Importantly, the source of protein appears to matter; plant-based proteins were associated with reduced mortality risk, while animal-based protein was linked to increased risk [69]. Short-term intervention trials in humans further support the health benefits of protein restriction. For instance, randomized controlled studies have shown that reducing protein intake improves glucose homeostasis [70]. A recent study by Nicolaisen et al. demonstrates that when lean and healthy men followed a low-protein diet (meeting minimum requirements) for 5 weeks, they tended to consume more calories, either from fats or carbohydrates, to maintain their body weight. Intriguingly, the protein-restricted diet improved whole-body insulin sensitivity when proteins were replaced by carbohydrates, which may involve FGF21 (fibroblast growth factor 21) [71].

### Intermittent fasting

Intermittent fasting (IF) has gained considerable attention in recent years, not only as a weight management strategy but also for its potential effects on longevity and healthspan. Intermittent fasting refers to eating patterns that cycle between periods of eating and fasting. Common approaches include alternate-day fasting (ADF) and periodic fasting (PF). The emerging time-restricted fasting (TRF) may also be considered as an alternative form of IF. TRF limits daily food consumption to a specific time window (e.g., 8 h) and fasting for the rest of the day. ADF alternates between regular eating days and fasting days with little or no caloric intake. PF involves restricting calories or complete fasting for longer periods (e.g., 2–5 days) at regular intervals.

Compelling evidence for the longevity-promoting effects of IF comes from research in various model organisms. In organisms such as worms [72] and flies [73, 74], intermittent fasting has been shown to extend lifespan by 20–40%. A variety of IF regimens, including ADF, PF, and TRF, have been tested in mice and rats, yielding robust evidence that IF can enhance longevity and delay the onset of age-related pathologies [75–78]. One of the earliest studies by Goodrick et al. demonstrated that ADF significantly extended both median and maximum lifespan in several strains of mice. These benefits were observed even when fasting was initiated in mid-life, suggesting that IF can be beneficial even if not practiced lifelong. Subsequent studies confirmed that rodents subjected to IF exhibit increased lifespan comparable to those under caloric restriction (CR), although the degree of extension varies with strain, sex, and fasting protocol [79, 80]. IF in rodents consistently results in improved insulin sensitivity, lower fasting glucose and insulin levels, and protection against high-fat diet-induced obesity [81–84]. Rodent studies have also shown that IF reduces the incidence and progression of age-related cancers and neurodegenerative diseases [27, 85–87]. Notably, several studies also indicate that the beneficial effects of IF are not attributable to reduced calorie intake [81, 84, 88]. It is also worth noting that the CR regimens implemented in early rodent studies were often associated with TRF, as the animals were fed once daily and consumed most of their meal within a short timeframe.

Although conclusive evidence that intermittent fasting (IF) extends human lifespan remains absent, numerous surrogate endpoints and health-related outcomes suggest that IF may provide longevity benefits [89–91]. IF elicits significant metabolic improvements. A controlled trial conducted by Sutton et al. in prediabetic men found that limiting food intake to a 6-h window improved insulin sensitivity, even in the absence of weight loss [92]. Complementary findings from Wilkinson et al. showed that 10-h TRF reduced fasting glucose levels and lowered HbA1c (Hemoglobin A1c) in individuals with metabolic syndrome [93]. In the same study, TRF also improved cardiovascular outcomes, such as blood pressure and atherogenic lipid levels, in patients. Recent findings by Kapogiannis et al. indicate that IF enhances executive function and memory while also attenuating the rate of brain aging in older adults [94].

## The underlying molecular and cellular mechanisms of dietary restriction

### Metabolic reprogramming

At the cellular level, DR promotes a transition from glucose-dependent metabolism toward alternative fuel utilization pathways. Different DR regimens, including CR, PR, and IF, trigger overlapping yet distinct metabolic adaptations. Previous studies in *C. elegans* and *Drosophila* demonstrate that CR leads to a metabolic shift toward fatty acid metabolism [95, 96], enhancing the organism’s ability to utilize stored lipids for energy production. Similarly, elevated lipogenesis and lipolysis are observed in mice subjected to CR or IF [97, 98], indicating that dynamic remodeling of lipid metabolism is needed to maintain metabolic homeostasis during periods of nutrient scarcity. It is worth noting that PR also influences lipid metabolism. In flies fed a high-fat diet, PR has been shown to alter lipid metabolic pathways [99]. Dietary methionine restriction persistently increases total energy expenditure and enhances metabolic flexibility while increasing uncoupled respiration in both fed and fasted states [100]. Enhanced fatty acid oxidation has long been linked to various longevity pathways [101, 102]. Research in *C. elegans* and *Drosophila* has also demonstrated that genetic upregulation of lipolytic pathways and fatty acid β-oxidation contributes to lifespan extension [103, 104], suggesting that a metabolic shift toward more efficient energy utilization may be critical for the longevity benefits.

IF, which involves cycling between periods of feeding and fasting, also leads to elevated ketogenesis, which is not found in CR or PR. Growing evidence supports the health benefits of ketone bodies in animal models [105, 106]. However, Tomita et al. found that ketone body supplementation may produce opposing effects, depending on the timing of administration. Ketone body supplementation prolongs lifespan when given to aged mice but increases mortality when administered early in life [107]. These findings suggest that the broader metabolic reprogramming caused by IF may have a greater impact on promoting longevity than the elevation of ketone bodies alone.

### Nutrient-sensing signaling networks

#### Insulin/IGF-1 signaling (IIS)

**Fig. 1:**
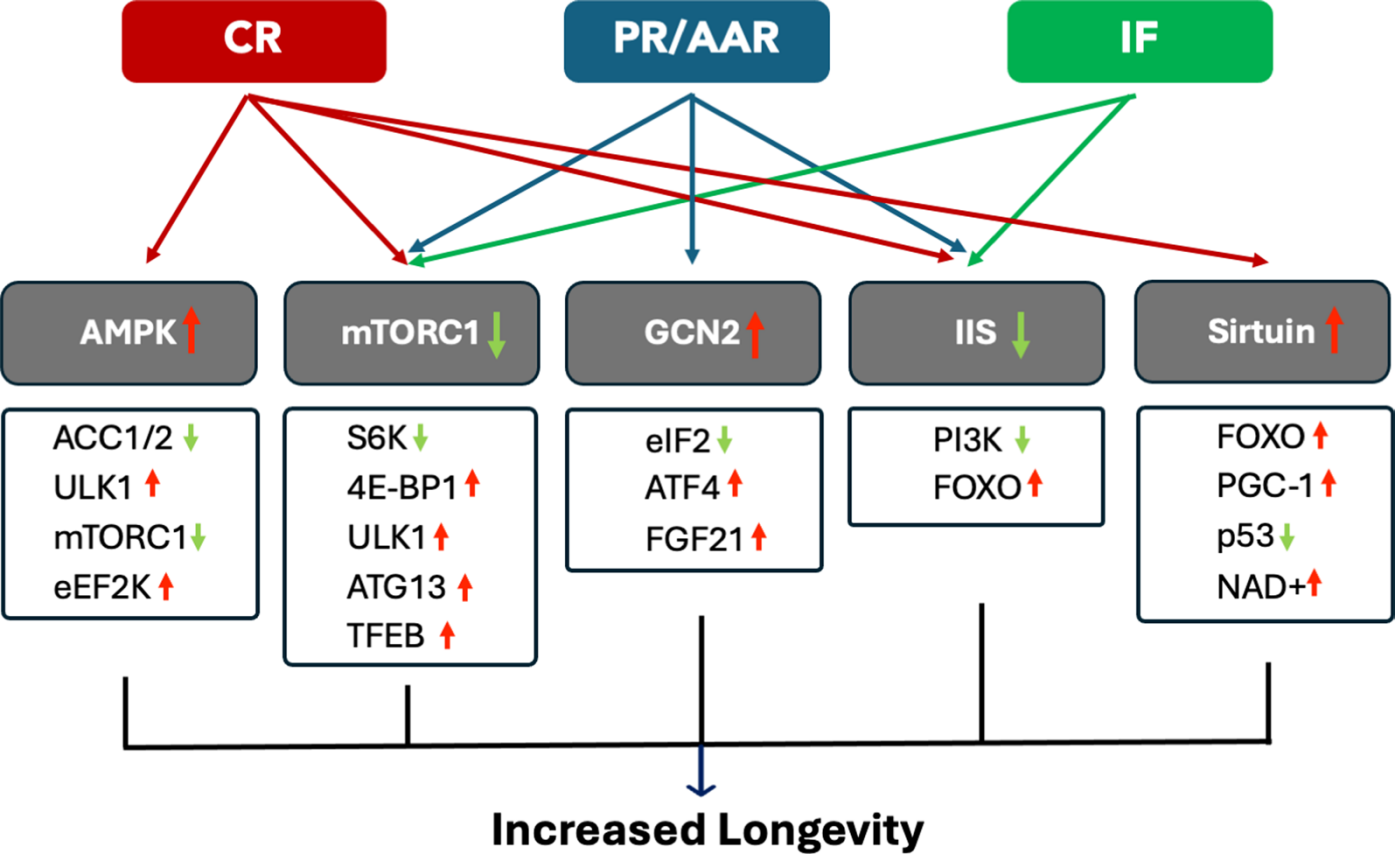
A schematic illustration summarizes the signaling pathways discussed in the text that might mediate the longevity effects of different DR regimens. Key components of each signaling pathway and their downstream effectors are included here. We list only the most recognized names of their mammalian homologs. The arrows indicate whether the activities or the levels of these key components are up- or down-regulated in response to different DR regimens

The insulin/IGF-1 signaling (IIS) is a highly conserved signaling pathway that regulates both metabolism and lifespan (Fig. 1). Reduced IIS activity and subsequent activation of pro-longevity FOXO (forkhead box O) transcription factor have been consistently demonstrated to extend lifespan in model organisms such as worms, flies, and mice [108, 109]. Accumulating evidence from vertebrate models or humans has suggested that CR leads to systemic decreases in glucose and insulin, thereby improving insulin sensitivity [37, 110]. Remarkably, PR triggers a similar metabolic benefit even in the absence of calorie reduction. In healthy individuals, PR has been shown to reduce blood glucose and insulin levels [111]. Furthermore, methionine restriction can mimic many of the effects of CR, including reduced blood glucose and insulin [112]. Similar effects in systemic glucose homeostasis could also be found in IF animals [81]. Therefore, the DR-induced longevity benefits may be mediated by inactivating the IIS pathway in vertebrates. Consistent with this model, studies in rodents have shown that the FOXOs become activated under DR conditions and are required for DR-mediated lifespan extension [113, 114].

#### mTORC1 signaling

The mammalian target of rapamycin (mTOR) pathway serves as a central hub for integrating signals from multiple nutrients and growth factors [115]. mTOR exists in two distinct complexes: mTORC1 and mTORC2, with mTORC1 being the primary nutrient sensor. When activated, mTORC1 promotes anabolic processes essential for cell growth and proliferation, including protein synthesis, lipid synthesis, nucleotide biosynthesis, and glycolysis. Conversely, mTORC1 activation suppresses catabolic processes such as autophagy and lysosomal biogenesis. Accumulating studies have established mTORC1 inhibition as an evolutionarily conserved mechanism for lifespan extension [116]. Pharmacological inhibition of mTORC1 with rapamycin also promotes health and longevity in diverse model organisms [117], making it an attractive target for anti-aging therapy.

Given the role of mTORC1 as a cellular nutrient sensor, DR is anticipated to suppress mTORC1 activity by reducing nutrient availability. Indeed, this has been demonstrated across multiple experimental paradigms, with CR, PR, and IF showing significant decreases in mTORC1 signaling activity in various tissues of mouse model [118–122]. mTORC1 regulates longevity mainly through modulating both protein synthesis and autophagy [116], two critical processes for protein homeostasis. When nutrients are abundant, mTORC1 drives cellular growth by activating translation regulators such as S6K1 and 4E-BP1, stimulating protein synthesis. In response to DR, reduced mTORC1 activity leads to a decrease in protein synthesis. The significance of this regulation is further highlighted by recent studies showing that genetic reduction of protein synthesis alone can extend lifespan [123]. mTORC1 also suppresses autophagy by suppressing the autophagy initiators, such as ULK1 and ATG13 (Autophagy related 13). Under DR conditions, reduced mTORC1 activity alleviates this inhibition, boosting autophagy for clearing damaged cellular components. Additionally, mTORC1 inhibition activates TFEB, a transcription factor that boosts lysosomal biogenesis and autophagy-related gene expression during nutrient scarcity. This elevating autophagy further promotes longevity, as impaired autophagy has been shown to block the health benefits of DR [124, 125].

#### AMPK signaling

AMP-activated protein kinase (AMPK) is a highly conserved serine/threonine kinase that functions as a central regulator of cellular energy homeostasis, activated in response to energy stress to restore ATP levels by modulating metabolic pathways [126]. Under DR conditions, reduced nutrient availability leads to decreased ATP levels and increased AMP/ATP ratios, which directly activate AMPK. Research in *C. elegans* demonstrates that AMPK activity is required for DR-induced longevity [127, 128]. Furthermore, genetic overexpression of AMPK extends lifespan in worms and flies [129, 130]. In rodents, DR activates AMPK in various tissues, including the heart and skeletal muscle [131–133]. Upon activation, AMPK triggers several longevity-promoting cellular mechanisms, such as autophagy. AMPK promotes autophagy by phosphorylating ULK1 (Unc-51-like kinase 1) and inhibiting mTORC1 [134]. The negative regulation of mTORC1 signaling by AMPK also results in decreased protein translation. Moreover, AMPK activates eukaryotic elongation factor 2 kinase (eEF2K), which suppresses translation elongation, thereby further reducing global protein synthesis [135]. Furthermore, the AMPK-eEF2K axis has also been implicated in the regulation of stress granule formation, which also contributes to DR-induced longevity in worms [128]. AMPK regulates lipid metabolism by phosphorylating acetyl-CoA carboxylase 1/2 (ACC1/2), thereby inhibiting the production of malonyl-CoA, a critical substrate for fatty acid synthase and a precursor for de novo palmitate synthesis. Since malonyl-CoA potently inhibits mitochondrial carnitine palmitoyl transferase 1 (CPT1) and thus limits fatty acid β-oxidation, AMPK-mediated ACC phosphorylation simultaneously suppresses lipogenesis while promoting fatty acid oxidation [126], a metabolic pattern typically observed in animals under DR.

#### Sirtuin signaling

Sirtuins are evolutionarily conserved enzymes that catalyze the removal of acetyl groups from lysine residues on target proteins. This reaction produces nicotinamide, O-acetyl-ADP-ribose, and a deacetylated protein, linking sirtuin activity to cellular NAD⁺ levels and metabolic state. The mammalian sirtuin family comprises seven members (SIRT1-7) with distinct subcellular localizations and substrate specificities [136]. SIRT1 emerges as the most extensively studied sirtuin in the context of DR and longevity regulation. In yeast, overexpression of Sir2, the homolog of mammalian SIRT1, extends lifespan by approximately 30% [137]. The role of Sir2/SIRT1 in mediating DR-induced longevity has been well-demonstrated in worms and flies, and mice as well [138–140], underscoring the evolutionary significance of sirtuins in lifespan regulation. Moreover, upregulation of SIRT1 expression has been observed in mice subjected to CR and fasting [140, 141], further supporting its involvement in nutrient-responsive pathways. While whole-body SIRT1 overexpression protects against metabolic disorders without extending lifespan, brain-specific overexpression of SIRT1 significantly increases lifespan and delays aging-associated decline [142, 143], suggesting that SIRT1 activity in the central nervous system might play a critical role in regulating aging. DR-induced activation of SIRT1 leads to the deacetylation of various transcription factors and metabolic regulators such as PGC-1α, FOXO proteins, and p53 [144]. This activation promotes a transcriptome-wide shift, contributing to improved cellular function and stress resistance. Supporting this, Levin et al. demonstrated that the effects of TRF-CR on hepatic gene expression in mice are largely mediated by an increase in the daytime peak of hepatic NADH levels, which inhibit SIRT1 activity to modulate transcriptional and metabolic responses [145]. Besides SIRT1, SIRT6 shares several pro-longevity attributes with SIRT1, including involvement in genome stability, epigenetic regulation, and attenuation of inflammation [146–148].

As sirtuin activity requires NAD^+^, the contributions of sirtuin and NAD^+^ metabolism for the beneficial effects of DR are difficult to separate. Thus, it is not surprising to find that the DR-induced lifespan extension requires both Sir2 and NAD^+^ synthesis pathway in yeast [149]. Altering key enzymes involved in the NAD^+^ metabolism, such as PNC1 or NAMPT, has been shown to affect lifespan in yeast, worms, and mice [150–153]. Consistent with this idea, nutritional supplementation with NAD^+^ or its precursors has been proposed to promote healthy aging, potentially via sirtuin activation [154].

#### The GCN-ATF4-FGF21 axis

GCN2 (general control nonderepressible 2) is a conserved serine/threonine kinase that also acts as an amino acid sensor to regulate various cellular and physiological responses, including immune system homeostasis and integrated stress responses [155–157]. Activation of GCN2 results in the phosphorylation of eIF2 (eukaryotic translation initiation factor 2), which inhibits the translation of most mRNA, while selectively upregulating the translation of certain proteins, including ATF4 (activating transcription factor 4) [158–160]. ATF4 is required for the elevated expression of several PR or AAR-responsive genes, including the hormone FGF21 (fibroblast growth factor 21) [161]. The roles of GCN2 in DR have been reported in both worms and rodents. In worms, *gcn-2* is required for the lifespan extension by the genetic model of CR (i.e., *eat-2* mutants) and TOR inactivation [162]. In rodents, *Gcn2-*knockout mice subjected to PR demonstrate a delayed metabolic response adaptation in response to reduced protein intake [163].

The FGF21 is a liver-derived hormone that has been implicated in many metabolic effects of PR, such as increased insulin sensitivity [164, 165]. It has also been reported that both the hepatic expression and circulating level of FGF21 are elevated in response to chronic PR in both rodents and humans [70, 164]. Moreover, mice lacking *fgf21* are less responsive to PR [164, 166]. The most compelling evidence came from the mouse studies, in which Zhang et al. have shown that FGF21 overexpression could extend mouse lifespan [167] and Hill et al. reported that FGF21 is required for PR to extend lifespan in male mice [168].

## Translational perspectives

The translation of DR research to human anti-aging interventions faces fundamental obstacles rooted in the complexity of human biology and lifestyle factors. While controlled studies in model organisms demonstrate clear benefits, human studies present unique challenges. Long-term adherence to restrictive eating patterns, individual genetic variability, and the influence of social and environmental factors all complicate the direct translation of laboratory findings. Recent clinical trials have shown promising results for IF and CR protocols in humans [37, 169], with observed improvements in insulin sensitivity, cardiovascular markers, and inflammatory profiles. However, the optimal timing (i.e., the age of the participant), duration, and intensity of dietary restriction interventions remain poorly defined.

Understanding the molecular mechanisms underlying DR benefits has opened new therapeutic avenues beyond traditional DR limitations. The identification of key signaling pathways, including mTOR, AMPK, and sirtuins, has led to the development of DR mimetics. These compounds aim to activate the same beneficial cellular responses as DR without requiring actual food limitation, potentially offering more practical alternatives for clinical application.

Rapamycin has emerged as one of the most promising DR mimetics through its specific inhibition of mTORC1. Rapamycin recapitulates many of the longevity benefits observed in DR animals, including enhanced autophagy, improved protein quality control, and increased stress resistance [170]. Clinical studies with rapamycin have demonstrated remarkable results, including improved immune function in elderly populations and enhanced vaccine responses [171]. However, chronic rapamycin use presents challenges, including increased risk of infections, delayed wound healing, and metabolic complications [172]. Recent research has focused on developing rapamycin analogs and intermittent dosing regimens that might maximize longevity benefits while minimizing adverse effects [173, 174].

Metformin, the world's most widely prescribed diabetes medication, has gained even more attention as a potential anti-aging intervention due to its ability to activate AMPK [175]. Large-scale epidemiological studies have shown that diabetic patients taking metformin often exhibit lower rates of age-related diseases, including cardiovascular disease [176], cancer [177, 178], and neurodegenerative disorders [179, 180], compared to those on other diabetes medications. Those observations have led to the TAME (Targeting Aging with Metformin) study, which aims to demonstrate metformin's potential as a geroprotective agent in non-diabetic populations [181]. Metformin's excellent safety profile, established through decades of clinical use, makes it a promising candidate for long-term use in non-diabetic or healthy populations.

The complementary mechanisms of rapamycin and metformin have sparked interest in combination approaches that target multiple pathways simultaneously. Preliminary studies in model systems suggest that combination therapy might provide additive or synergistic benefits [182–184], potentially allowing for lower doses of each drug while maintaining efficacy. The new strategies represent a compelling intersection of basic science discovery and clinical application.

It is worth noting that while DR has been regarded as the most robust intervention to promote health and increase lifespan, it does come with some trade-offs. The most commonly known trade-off is reduced fecundity [185–187]. However, some studies suggested that longevity and reproduction may be uncoupled under certain DR conditions [187, 188]. Furthermore, it has been reported that men and populations with elevated BMI who undergo DR may have mental health consequences, such as increased depressive symptoms [189]. Therefore, the use of DR mimetics as an anti-aging intervention in humans should still be cautious before more clinical studies are completed.

## Conclusion

The overlapping yet distinct mechanisms of different DR regimens, including CR, PR, AAR, IF, and TRF, offer us numerous potential targets for future translational research. As our understanding of the underlying mechanisms of these DR regimens continues to grow, the development of safe and effective interventions that mimic the benefits of DR holds significant promise for promoting healthy aging and preventing age-related diseases in human populations.

## Data Availability

No datasets were generated or analysed during the current study.
